# P-cadherin counteracts myosin II-B function: implications in melanoma progression

**DOI:** 10.1186/1476-4598-9-255

**Published:** 2010-09-22

**Authors:** Koen Jacobs, Mireille Van Gele, Ramses Forsyth, Lieve Brochez, Barbara Vanhoecke, Olivier De Wever, Marc Bracke

**Affiliations:** 1 Laboratory of Experimental Cancer Research, Department of Radiation Oncology and Experimental Cancer Research, Ghent University Hospital, Ghent University, De Pintelaan 185, Ghent, 9000, Belgium; 2 Department of Dermatology, Ghent University Hospital, Ghent University, De Pintelaan 185, Ghent, 9000, Belgium; 3 Department of Pathology, Ghent University Hospital, Ghent University, De Pintelaan 185, Ghent, 9000, Belgium

## Abstract

**Background:**

Malignant transformation of melanocytes is frequently attended by a switch in cadherin expression profile as shown for E- and N-cadherin. For P-cadherin, downregulation in metastasizing melanoma has been demonstrated, and over-expression of P-cadherin in melanoma cell lines has been shown to inhibit invasion. The strong invasive and metastatic nature of cutaneous melanoma implies a deregulated interplay between intercellular adhesion and migration-related molecules

**Results:**

In this study we performed a microarray analysis to compare the mRNA expression profile of an invasive BLM melanoma cell line (BLM LIE) and the non-invasive P-cadherin over-expression variant (BLM P-cad). Results indicate that nonmuscle myosin II-B is downregulated in BLM P-cad. Moreover, myosin II-B plays a major role in melanoma migration and invasiveness by retracting the tail during the migratory cycle, as shown by the localization of myosin II-B stress fibers relative to Golgi and the higher levels of phosphorylated myosin light chain. Analysis of P-cadherin and myosin II-B in nodular melanoma sections and in a panel of melanoma cell lines further confirmed that there is an inverse relationship between both molecules.

**Conclusions:**

Therefore, we conclude that P-cadherin counteracts the expression and function of myosin II-B, resulting in the suppression of the invasive and migratory behaviour of BLM melanoma cells

## Background

Cutaneous melanoma, an aggressive cancer type originating from melanocytes in the human skin, is characterized as an invasive and commonly metastasizing tumor which is the major cause of death of melanoma patients [1,2] Normal cutaneous melanocytes form cell-cell contacts with adjacent keratinocytes, providing a molecular anchor by which melanocytes participate in the normal function and architecture of the human skin. Malignant transformation of melanocytes is featured by downregulation of cell-cell adhesion molecules like E- and P-cadherin, resulting in the loss of keratinocyte-mediated growth and motility control [3,4]. Concomitant with these changes, melanoma cells often undergo a phenomenon, referred to as epithelial-to-mesenchymal transition (EMT), and obtain a migratory and protease-producing phenotype, leading to invasion and the formation of distant metastasis [5,6]. P-cadherin is a calcium-dependent cell-cell adhesion molecule belonging to the cadherin superfamily which comprises transmembrane proteins grouped by the presence of one or more cadherin repeats in their extracellular domains. The classical cadherin family consists of E(pithelial)-, N(euronal)-, V(ascular)E(ndothelial)- and P(lacental)-cadherin, named after the tissue they were first identified in [7,8]. Classical cadherins exert cohesive and organising functions that are required for tissue development and integrity. In contrast to the universal expression of E-cadherin in epithelia, P-cadherin is only expressed in the basal layer of squamous epithelia [9,10]. In epithelial-derived cancer, cadherins are often downregulated resulting in decreased cellular cohesion, increased invasion and formation of metastasis [11]. Our group showed that stable introduction of P-cadherin in BLM melanoma cells inhibits invasive capacities, stimulates homo- and heterotypic adhesion and induces an epithelioid phenotype [12]. Other experimental work elucidating the functions of P-cadherin pointed out that this molecule can have an opposite role depending on the cellular context [9,12-14]. Non-muscle myosin II belongs to the myosin superfamily and is an ATP-dependent molecular motor protein that can interact with and contract filamentous actin (F-actin) [15]. In vertebrates, three isoforms of the non-muscle myosin II heavy chain have been described: II-A, II-B and II-C. Each isoform is encoded by a specific gene and despite considerable homology between the different isoforms, differences in subcellular localization, enzymatic properties, filament assembly-disassembly regulation and tissue expression patterns have been described [16]. Nonmuscle myosin II heavy chain B (myosin II-B) plays a major role in the retraction phase of the migratory cycle in contrast to the bipolar shape- and substrate adhesion-related protrusion functions of nonmuscle myosin II heavy chain A (myosin II-A) [17-19]. In migrating endothelial cells, it has been shown that myosin II-A is more abundant near the leading edge and myosin II-B in trailing ends. Moreover, myosin II-A moves with a higher velocity into new protrusions whereas myosin II-B is confined much longer to the retraction site of the migrating cell [20]. Myosin II-B filament assembly and ATPase activity are regulated by phosphorylation via two main kinases, myosin light chain kinase and Rho kinase, which phosphorylate Ser19 at the regulatory light chain [21-23].

We show here that the anti-migratory and anti-invasive capacity of P-cadherin in BLM melanoma cells can be related to P-cadherin-dependent downregulation and organization of the myosin II-B isoform, implicating a coordinated cross-talk between adhesion molecules and cellular migration-related proteins.

## Results

### Myosin II-B is downregulated in BLM cells overexpressing P-cadherin

**Figure 1:**
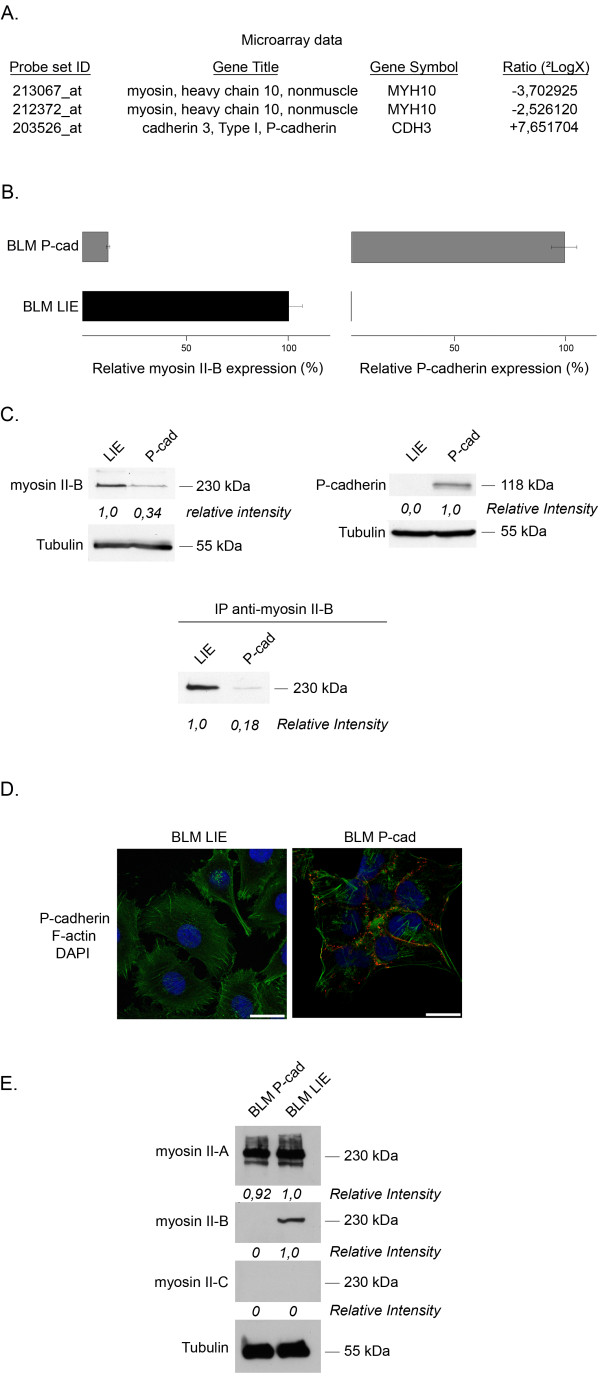
**P-cadherin and myosin II-B expression in BLM LIE versus BLM P-cad**. **(A) **Nonmuscle myosin heavy chain 10 (myosin II-B) mRNA was downregulated in BLM P-cad cells according to microarray analysis. Cadherin 3 (P-cadherin) mRNA levels were, as expected, upregulated in BLM P-cad. **(B) **Quantitative PCR validated the microarray data, indicating a 90% (± 5%) decrease in myosin II-B RNA expression in BLM P-cad and no P-cadherin RNA levels in BLM LIE. Error bars show the standard deviation for 2 independent experiments. **(C) **Decreased myosin II-B protein expression in BLM P-cad verifies microarray and PCR data. **(D) **Immunofluorescence staining of P-cadherin indicates the presence of functional P-cadherin mediated cell-cell contacts in BLM P-cad. **(E) **Western blot of myosin II isoforms in BLM LIE and P-cad. Equal amounts could be detected for myosin II-A, downregulation in BLM P-cad for myosin II-B, and no expression levels for myosin II-C. *(scale bar = 25 μm)*

Our group showed that stable introduction of P-cadherin in BLM melanoma cells induced major morphological (epithelioid phenotype) and functional differences (adhesion, invasion) between the two cell lines [12]. A microarray experiment performed with Affymetrix^® ^chips allowed us to detect transcriptional differences between BLM LIE and BLM P-cad cells. Figure 1A demonstrates that P-cadherin mRNA was strongly upregulated in BLM P-cad in contrast to BLM LIE cells, as expected, and that myosin II-B was downregulated in BLM P-cad cells (GEO Accession Number GSE23360). The results obtained from the microarray analysis were confirmed by quantitative polymerase chain reaction (qPCR). Normalised amounts of myosin II-B mRNA were 12,39% versus 100% for BLM P-cad and BLM LIE, respectively (figure 1B). Also on the protein level, downregulation of myosin II-B could be detected in BLM P-cad (figure 1C). Furthermore, the presence of membranous P-cadherin in BLM P-cad cells was validated (figure 1D). Since myosin II-B belongs to the nonmuscle myosin II family, which also contains closely-related myosin II-A and II-C isoforms, we wanted to ensure that myosin II-B was specifically downregulated in BLM P-cad cells without an effect on II-A and II-C expression. Figure 1E shows that indeed myosin II-B was specifically downregulated in BLM P-cad. We chose to further investigate myosin II-B because of the statistically significant results of the microarray and qPCR data and its contribution to cell migration as pointed out by current scientific literature.

### Myosin II-B co-localizes with F-actin bundles and functions at the trailing end of migrating BLM cells

**Figure 2:**
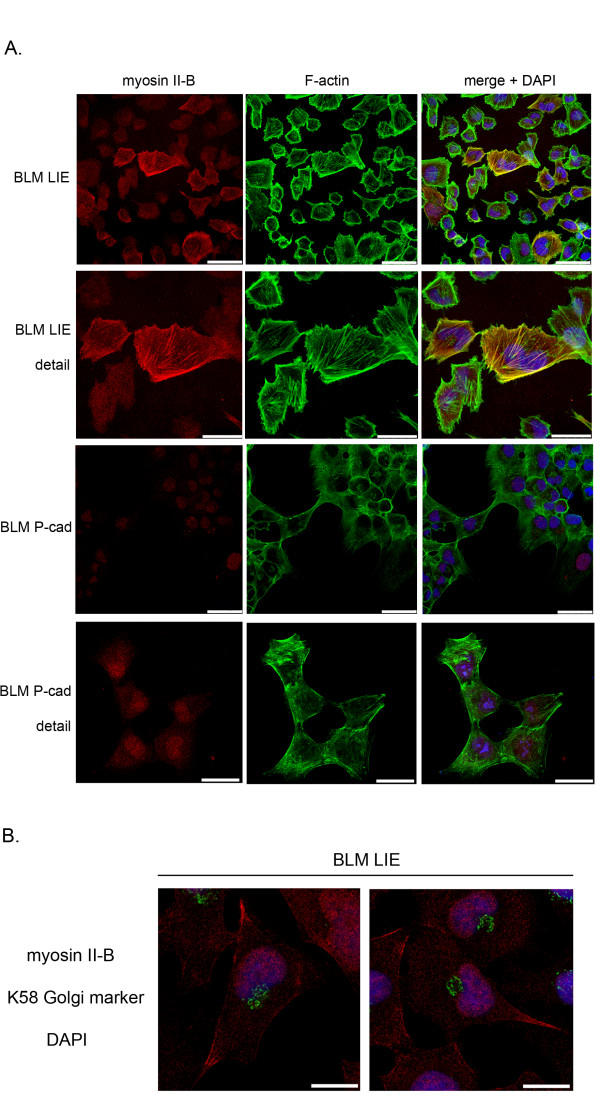
**Confocal images of myosin II-B in BLM LIE and BLM P-cad**. **(A) **Myosin II-B (red) confocal images with the F-actin cytoskeleton (green) and merge with DAPI (blue) nuclear staining. The detailed images show the formation of myosin II-B stress fibers and colocalization with F-actin, as indicated in the merge by yellow coloured stress fibers, in BLM LIE. Note the weak and diffuse myosin II-B staining pattern in BLM P-cad (*scale bar = 50 μm, for detail image scale bar = 25 μm*). **(B) **Immunofluorescence double staining of myosin II-B (red) and K58 Golgi protein (green). Position of Golgi and myosin II-B relative to nucleus (blue) reveals the function of myosin II-B occurring at the trailing end (*scale bar = 25 μm)*.

To provide information of myosin II-B filament assembly and subcellular localization, we performed immunofluorescence staining of nonmuscle myosin II-B. Figure 2A shows myosin II-B stress fiber formation in BLM LIE cells in contrast to the faint and diffuse distribution of myosin II-B in BLM P-cad cells. A double staining of myosin II-B and F-actin shows co-localization of the myosin stress fibers with F-actin bundles in BLM LIE cells. Note that myosin II-B stress fiber formation is not homogenous in BLM LIE cells, due to the fast and transient nature of myosin II function.

To determine whether myosin II-B stress fibers contribute to tail retraction in melanoma cells, we performed a double staining of myosin II-B and K58, a marker for the Golgi apparatus. It has been shown that the position of the Golgi and microtubule organizing centre faces the leading edge of a migrating cell. Immunofluorescence staining of the Golgi and myosin II-B therefore indicates whether the observed myosin II-B stress fibers are formed at the leading or trailing edge of the cell. Figure 2B shows myosin stress fibers at the retraction site of polarized BLM LIE cells.

### Higher activation status of myosin II-B in BLM LIE than in BLM P-cad

**Figure 3:**
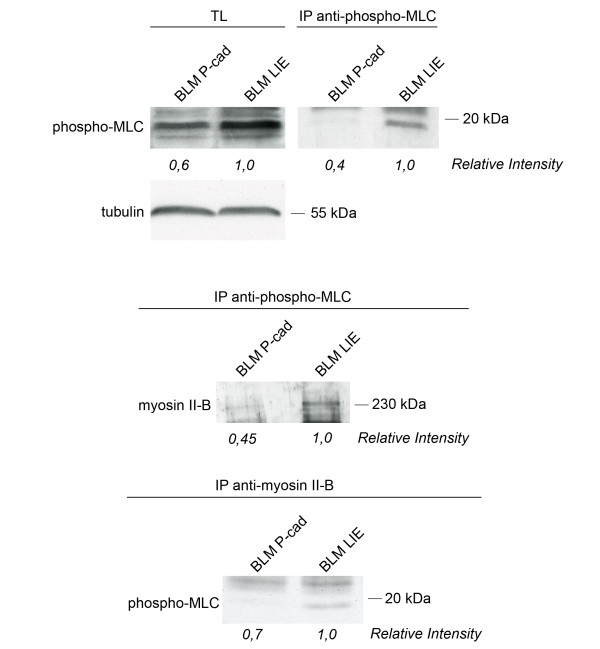
**Western blot to verify molecularly the activation status of MLC associated with myosin II-B**. The *upper panel *shows the levels of phosphorylated MLC in BLM LIE and BLM P-cad cells in total lysate (TL) and purified by immunoprecipitation. BLM P-cad shows a reduced amount of total phosphorylated MLC. *Lower panels *show the amount of phosphorylated MLC associated with myosin II-B by co-immunoprecipitation, indicating the decreased activation status of myosin II-B.

The activation status of nonmuscle myosin II can be characterized by phosphorylation of the regulatory light chain (MLC). This 20 kDa light chain contains two main phosporylation sites; Ser19 and Thr18. Myosin light chain kinase (MLCK) activity is necessary for the monophosphorylation at Ser19 of myosin light chain, thereby enhancing MgATPase activity and promoting myosin II filamentation. We show here that a higher level of total phosphorylated MLC can be detected in BLM LIE cells compared with BLM P-cad cells. When the phosphorylated MLC was precipitated, we stained for myosin II-B in order to detect the amount of phosporylated MLC that was specifically bound to myosin II-B. Figure 3 shows that low levels of activated nonmuscle myosin II-B could be detected in the non-invasive BLM P-cad cells in contrast to higher levels in invasive BLM LIE cells.

### Depletion of myosin II-B decreased cell migration and invasion

**Figure 4:**
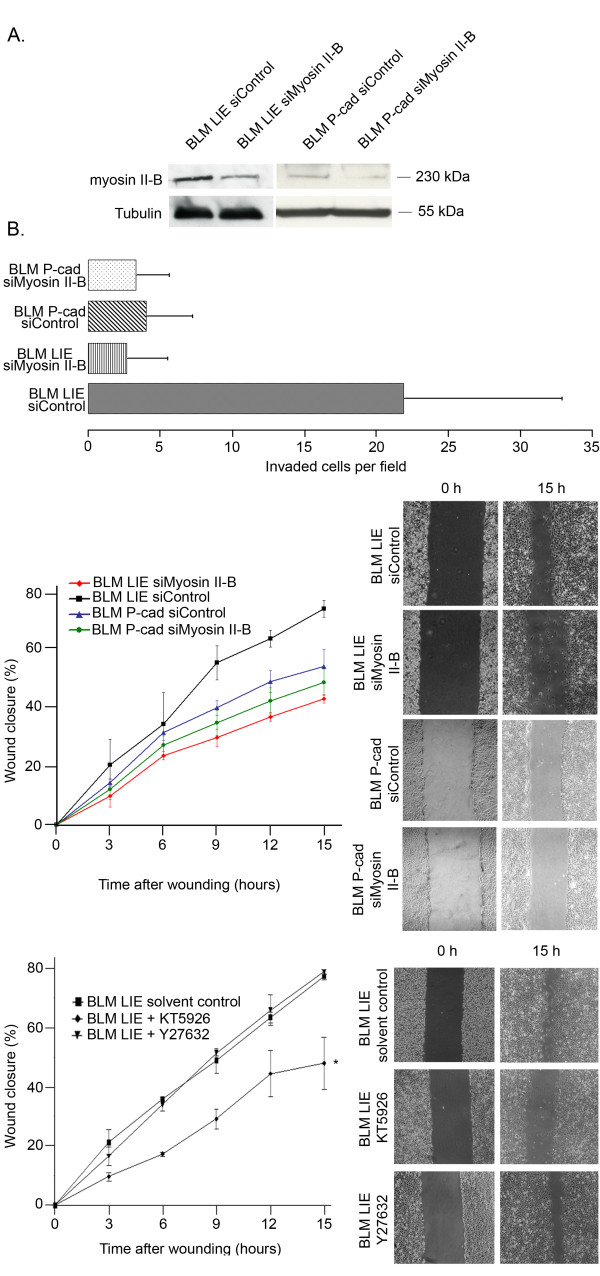
**Function of myosin II-B in BLM melanoma cell migration and invasion**. **(A) **Western blot to verify silencing of myosin II-B expression 72 h post-transfection in BLM LIE and BLM P-cad cells. Matrigel invasion assay shows reduced invasion of BLM LIE siMyosin II-B when compared to control cells (p < 0,0001). No significant difference could be observed for BLM P-cad siMyosin II-B compared to BLM P-cad siControl (p = 0,2). **(B) **Reduced migration of BLM LIE siMyosin II-B compared to BLM LIE siControl could be observed (p = 0,008) (*upper panel*). No significant difference between BLM P-cad siControl and siMyosin II-B could be detected (p = 0,7). Results of a wound healing assay with addition of specific kinase inhibitors (*lower panel)*. KT-5926 significantly reduced cellular migration when compared to control situation, in contrast to Y27632 (p = 0,009; p = 0,98).

Silencing of myosin II-B in BLM LIE cells resulted in decreased wound healing migration; after 15 h, 72% (± 2,8%) of the wound was closed with BLM LIE control cells whereas only 42% (± 1,4%) of the wound was closed with BLM LIE cells silenced for myosin II-B. For BLM P-cad cells, no significant difference could be detected between control cells and cells silenced for myosin II-B. Wound closure after 15 hours incubation by BLM P-cad control cells and BLM P-cad siMyosin II-B was 52% (± 5,6) and 47% (± 4,4), respectively. Regarding to invasion, a significant reduction of invaded cells through matrigel could be observed for BLM LIE cells silenced for myosin II-B in contrast to BLM P-cad cells (figure 4A and 4B).

### Inhibiting MLCK activity reduces cellular migration

Since the main regulators of filament assembly and ATPase activity of myosin II are myosin light chain kinase (MLCK) and Rho kinase (Rock), we used specific inhibitors of MLCK (KT-5926) and Rock (Y-27632) to identify which regulatory pathway is mainly involved in the regulation of nonmuscle myosin II-regulated cellular migration of BLM cells. By applying the wound healing assay we found that in control situation, approximately 70% of the initial wound was closed. When cells were incubated with Y27632 no significant difference in wound closure could be detected. In contrast, after treatment with KT-5926, approximately 45% of the initial wound was closed (figure 4B).

### An inverse relationship between P-cadherin and myosin II-B expression in a panel of melanoma cell lines

**Figure 5:**
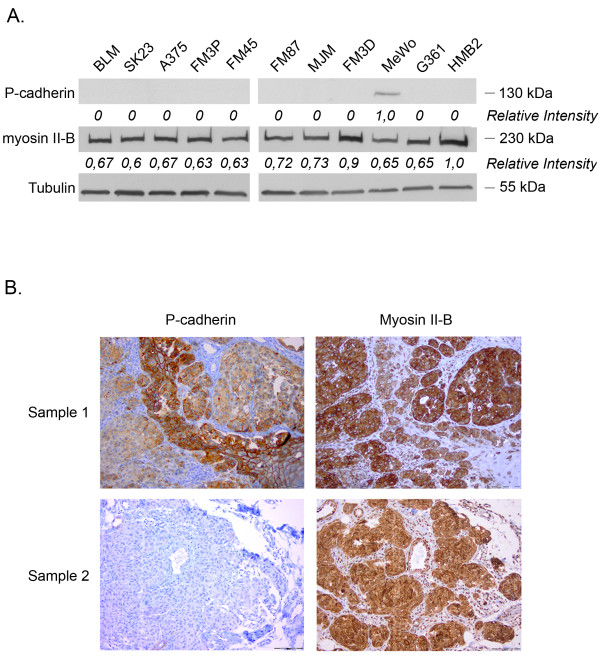
**(A) Western blot to determine the relative amounts of P-cadherin and myosin II-B in a series of melanoma cell lines**. High expression levels of myosin II-B for all melanoma cell lines, and absent or low expression levels of P-cadherin could be detected. **(B) **Immunohistochemical staining of myosin II-B and P-cadherin in nodular melanoma sections (NM). P-cadherin staining shows a honeycomb pattern in normal epidermal layers in contrast to tumor sites. The faint and diffuse staining pattern of the tumor sites indicates loss of cellular cohesion. Myosin II-B staining demonstrates strong positivity at tumor sites, whereas no staining could be observed in normal (epi)dermal layers.

Myosin II-B and P-cadherin expression levels were determined in 11 melanoma cell lines thereby expanding the hypothesis based on BLM cells to a wider panel of melanoma cell lines. As shown in figure 5A, all melanoma cell lines, except MeWo, were negative for P-cadherin and staining for myosin II-B reveals a high expression level in all melanoma cell lines.

### Nodular melanoma sections showed cytoplasmic staining for myosin II-B and an aberrant or absent P-cadherin expression at the nodular tumor site

To support the hypothesis that P-cadherin-mediated cellular cohesion and the expression and/or organisation of myosin II-B inversely correlate in malignant melanoma and contribute to invasion, we performed P-cadherin and myosin II-B stainings on eight nodular melanoma patient samples. Staining for P-cadherin showed a honeycomb-like pattern in the normal epidermal layers. In contrast, the tumor sites showed an aberrant or absent P-cadherin expression, indicating diminished cellular cohesion, which is an important aspect of invasion. Alternately, stainings of adjacent section for myosin II-B showed cytoplasmic staining in the tumor sites. Figure 5B presents two representative patient samples stained for P-cadherin and myosin II-B.

## Discussion

The formation of metastasis is the main cause of mortality of cancer patients. These processes require an initial, crucial step, namely invasion of tumor cells in surrounding stroma, where loss of intercellular adhesion and the gain of a migratory phenotype are of major importance. Differentiating basal cells migrate perpendicularly to the basal layer towards the surface of the skin, and various established cell-cell contacts maintain the epithelial structure and promote coordinated cellular migration. However, melanoma has been described as a strong invasive and metastasizing tumor, implicating loss of cellular cohesion and simultaneously acquirement of a migratory phenotype. Loss of cohesion has been evidenced by showing a cadherin shift in melanocytic tumors leading to decreased homo- and heterotypical intercellular adhesion and providing molecular anchors to interact with vascular endothelial cells and fibroblasts [24,25]. The close relationship between cell-cell adhesion proteins with polarity- and migration-related proteins, both in normal tissue and tumor, implicates a coordinated cross-talk between cell-cell adhesion molecules and migration-related proteins. This interaction has been shown between E-cadherin and polarity- and migration-related proteins like Rho GTPases. However, in most cases signal transduction pathways are pointed out, in contrast to the functional effector proteins [26,27]. In this paper, we describe that the expression of P-cadherin counteracts the expression and organization of nonmuscle myosin II-B which results in the repression of the migratory and invasive behaviour of melanoma cells. Due to the initial observation that introduction of P-cadherin in invasive BLM melanoma cells promoted cell-cell adhesion and counteracted invasion [12], we tried to determine molecular targets of P-cadherin that contribute to the anti-invasive and anti-migratory effect mediated by P-cadherin. Nonmuscle myosin II-B, implicated in cellular migration, cytokinesis and other cellular activities, could be identified as a functional effector protein that was downregulated in BLM P-cad cells. Myosin II-B staining of BLM LIE and BLM P-cad showed a stress fiber pattern in BLM LIE occurring at the trailing end of a polarized migrating cell, in contrast to BLM P-cad where no myosin II-B stress fibers, but a faint and diffuse, cytoplasmic expression pattern could be detected. Moreover, the localization of myosin II-B stress fibers relative to Golgi and the function of myosin II-B in cellular migration and invasion, demonstrates its contribution to these cellular activities by retracting the tail. Furthermore, the amount of phosphorylated regulatory light chain associated with myosin II-B is significantly higher in BLM LIE than in BLM P-cad. One of the morphological features of BLM P-cad cells is the formation of lamellopodia and filopodia but the tails of the cells are anchored by P-cadherin-mediated cell-cell contacts. Since the subcellular localization of both P-cadherin and myosin II-B occur at the same site of nucleus with reference to the Golgi apparatus, mutual and localized regulation can easily occur. All the tested melanoma cell lines showed no P-cadherin expression and high levels of myosin II-B. Interestingly, MeWo has been described as a non-invasive melanoma cell line and showed P-cadherin positivity. In order to confirm whether the P-cadherin switch in accordance with high myosin II-B expression effectively acts in melanoma patients, we evaluated nodular melanomas for P-cadherin and myosin II-B positivity, because of the deep infiltrative nature of this melanoma subtype. Normal epidermal layers showed a strong honeycomb-like P-cadherin staining, designating the cohesive nature of the normal skin. In contrast, the deeper tumor sites showed an aberrant or lack of expression of P-cadherin.

Cross-talk between adhesion molecules, e.g. E-cadherin, N-cadherin and αvβ3 integrin, and melanoma progression has been widely described. αvβ3 Integrin expression in human melanomas promotes invasion through physical interaction with MMP-2, and inducing transendothelial migration by association with the human neural cell adhesion molecule L1 expressed on endothelium [28]. For cadherins, loss of E-cadherin expression during melanoma progression has been shown to promote melanoma migration and, paralleled by N-cadherin upregulation, increased gap-junctional and N-cadherin-mediated communication with dermal fibroblasts and endothelial cells [29-31]. In this study, we present an important effector molecule that can be related to P-cadherin expression in invasive melanoma.

The molecular mechanism underlying P-cadherin-mediated myosin II-B regulation concerning migration and invasion will be the major focus for our future research. Our data suggest that mainly MLCK activity contributes to cellular migration in BLM melanoma cells. This means in our proposed model that introduction of P-cadherin in melanoma cells results in spatial MLCK inhibition at sites of P-cadherin-mediated cell-cell contacts. Since melanocytes originate from segregation of cell lineages derived from the cephalic neural crest, the neural genetic and epigenetic background may not be overruled [32]. Therefore, we hypothesize that, as shown in neuronal cells, cadherin expression can modulate calcium channels and thereby alter transient calcium influxes and available intracellular calcium at sites of cadherin-mediated cell-cell contacts, as several ion channels and ion channel regulated proteins are presented in the microarray list (Additional file 1). Directional migration of a neuron is the succession of three major processes: protrusion of the leading edge, cell body translocation and retraction of the trailing end [33]. For each of these processes, a critical, regulating role of calcium waves has been pointed out [34]. Important, Shengyu Yang et al. recently showed that calcium-influxes, rather than calcium release of intracellular stores, are critical for growth-factor induced cell migration. Moreover, they describe that calcium channels facilitating calcium-influxes activate MLCK specifically at the trailing tail, without an effect on the phosphorylation status of myosin light chain at the lamellopodia, supporting our hypothesis [35]. An intensively studied calcium flux modulator is the NMDA receptor. Besides its presence in an 'industrial complex' associated with an enormous variety of cytoskeletal proteins (including myosin II-B), cellular adhesion proteins (including cadherins) and signaling proteins (including calmodulin kinase and tyrosine kinases) at the postsynaptic membrane for regulating calcium influx, it is a known regulator of cellular migration. Indeed, it has been shown that there is a positive correlation between the rate of cell movement and the amplitude and frequency of calcium fluctuations governed by NMDA receptors [36,37]. Therefore, it would be interesting to explore further the molecular link between cadherins, calcium signaling and myosin II activity and their implications in melanoma progression.

## Conclusions

In general, we can conclude that the inhibition of invasion and impaired migration of melanoma cells can, at least partially, be ascribed to a P-cadherin-mediated decreased myosin II-B expression and impaired organization. This interplay between adhesion and migration may be an important strategy of the melanoma cell to invade the deeper stroma and to form metastasis.

## Methods

### Plasmids, cDNA construction, retroviral transduction and cell sorting

Plasmid and cDNA construction, retroviral transduction and cell sorting of BLM melanoma cells containing p**L**ZRS-**I**RES-**E**GFP (BLM **LIE**) and p**P-cad**herin-IRES-EGFP (BLM **P-cad**) were obtained as described earlier [12].

### Human cell lines and culture conditions

Parental BLM melanoma cells were obtained from L. Van Kempen (University of Nijmegen, The Netherlands). Cells were grown and maintained in Dulbecco's modified Eagle's medium (DMEM) (Gibco BRL, Belgium) containing 10% heat-inactivated fetal bovine serum (Greiner Bio-One, Belgium), 100 IU per mL penicillin, 100 μg per mL streptomycin and 2,5 μg per mL amphotericin B. Other melanoma cell lines used include SK-23, A-375, FM3P, FM45, FM87, MJM, FM3 D, MeWo, G361 and HMB-2. SK-23, A-375, MJM, MeWo and HMB2 were maintained in DMEM (Gibco BRL, Belgium) containing 10% heat-inactivated fetal bovine serum (Greiner Bio-One, Belgium), and antibiotics. FM3P, FM3 D, FM45 and FM87 were maintained in Roswell Park Memorial Institute 1640 (RPMI 1640) (Gibco BRL, Belgium) and G361 cells were maintained in McCoy's 5A medium (Gibco BRL, Belgium).

### Antibodies

Rabbit polyclonal anti-nonmuscle myosin II-A, B and C (Cell Signaling Technology, MA, US) for Western blot and immunoprecipitation experiments, rabbit polyclonal anti-nonmuscle myosin II-B (Covance, CA, US) for immunofluorescence and immunohistochemistry, mouse monoclonal anti-58K Golgi protein (Abcam, UK), rabbit polyclonal anti-phospho-myosin light chain (pSer19) (Sigma, MO, US), monoclonal mouse anti-P-cadherin clone 56 (BD Biosciences, Belgium). Anti-α-tubulin antibody (Sigma, MO, US) was used as loading control for Western blot. Secondary antibodies used for Western blot were ECL™ anti-mouse IgG horseradish peroxidase linked whole antibody from sheep and ECL™ anti-rabbit IgG horseradish peroxidase linked whole antibody from donkey (GE Healthcare, UK). For immunostaining, Alexa Fluor 594 goat anti-mouse and anti-rabbit IgG and Alexa Fluor 488 goat anti-rabbit and anti-mouse IgG (Invitrogen, Belgium) were applied.

### Polyacrylamide gel electrophoresis (PAGE), Western blotting and immunoprecipitation experiments

Cells were lysed using PBS containing 1% Triton X-100, 1% NP40 (Sigma, MO, US) and the following protease inhibitors: Leupeptin (10 μg/mL), aprotinin (10 μg/mL) (ICN Biomedicals, CA, US), phenylmethylsulfonyl fluoride (1.72 mM), NaF (100 μM), NaVO3 (500 μM), and Na4P2O7 (500 μg/mL) (Sigma, MO, US). After preparing lysates, protein concentration was measured using Rc-Dc protein assay kit (Bio-Rad Laboratories S.A.-N.V, Belgium). For immunoprecipitation experiments, primary antibody was added to the supernatant and incubated for 3 h at 4° C followed by the addition of protein A- or G Sepharose (Amersham Pharmacia Biotech, UK) beads for 1 hour. Sample buffer (Laemmli) was added, sample was boiled and proteins were separated by electrophoresis and blotted onto a nitrocellulose membrane (Amersham Pharmacia Biotech, UK) and immunostained in PBS supplemented with 0,5% tween 20 (Duchefa Biochemie, The Netherlands) and 5% non-fat dry milk in PBS. Chemiluminescent substrate (ECL Western blotting detection reagent, GE Healthcare, Belgium) was added and a film was exposed. Films were scanned (Epson perfection 4990, Epson, Belgium) and bands were quantified using Quantity One^® ^software (Bio-Rad Laboratories S.A.-N.V, Belgium).

### Confocal imaging of myosin II-B, F-actin and 58K Golgi protein

For myosin II-B immunofluorescence staining, BLM cells were fixed in 3,7% formaldehyde in Cytoskeleton Stabilization Buffer (CSB) (NaCl (137 mM), KCl (5 mM), NA2HPO4.2H20 (1,1 mM), KH2PO4 (0,4 mM), MgCl2.6H20 (2 mM), EGTA (2 mM), PIPES (5 mM) and D-Glucose (5,5 mM) dissolved in desionised water). Fixed cells were permeabilized with 0,5% Triton X-100 (Bio-Rad Laboratories, Belgium) in CSB. Cells were treated with 50 mM NH4Cl and blocked with 5% albumin from bovine serum (Sigma, MO, US). Afterwards, primary antibody was applied for 1 hour on room temperature and the secondary antibody for 30 minutes. For F-actin/myosin II-B double staining, a mixture of 15 μL phalloidin-fluorescein isothiocyanate (20 mM) (Sigma, MO, US), 585 μL phosphate-buffered saline, 3 μL MgSO4 (1 M) and 30 μL ethylene glycol tetraacetic acid (0,1 M) was combined with the anti-rabbit secondary antibody.

### RNA isolation, microarray analysis and quantitative PCR

RNA isolation was performed using an RNeasy mini-kit (Qiagen Benelux, The Netherlands) according to manufacturer's instructions. RNA concentration and purity were determined spectrophotometrically using the Nanodrop ND-1000 (Nanodrop Technolgies, DE, US) and RNA integrity was assessed using a Bioanalyser 2100 (Agilent, CA, US). Per sample, an amount of 2 μg of total RNA spiked with bacterial RNA transcript positive controls (Affymetrix, UK) was converted and amplified to double stranded cDNA in a 1-cycle cDNA reverse transcription reaction. Subsequently the sample was converted to antisense cRNA and labeled with biotin through an *in vitro *transcription reaction according to the manufacturers protocol (Affymetrix, UK). All amplification and labelling reactions were performed on a Biomek 3000 ArrayPlex Workstation (Beckman Coulter, Belgium). A mixture of purified and fragmented biotinylated cRNA and hybridisation controls (Affymetrix, UK) was hybridised on Affymetrix HG U133 Plus 2.0 arrays followed by staining and washing in the GeneChip^® ^fluidics station 400 (Affymetrix, UK) according to the manufacturer's procedures. To assess the raw probe signal intensities, chips were scanned using the GeneChip^® ^scanner 3000 (Affymetrix, UK). Probe signal intensities were statistically analyzed by Student t-test and statistically significant results were checked by quantitative polymerase chain reaction (qPCR) using SYBR green method as described by M. Van Gele [38]. To quantify the gene expression levels of nonmuscle myosin II heavy chain B (MYH10) and P-cadherin (CDH3) transcripts, primer sequences were designed using Primer Express software (Applied Biosystems, CA, US) (MYH10 forward primer: CCTCATGCTGACCTTGCAAA - reverse primer: GGACACAAAACCAATATTCCCATT) (CDH3 forward primer: AGTGGAGGACCCCATGAACA - reverse primer: CTGGGTAAACTTGGGCTTGTG) A BLAST search was used to assure that these primers were specific for nonmuscle myosin II heavy chain B and P-cadherin. Relative gene expression levels were determined using a SYBR Green I reverse transcription-PCR assay as described by Vandesompele et al. and the comparative Ct method was used for quantification [39,40].

### Transfection of short interference RNA

Sense and antisense short interference RNA (siRNA) oligos for myosin II-B [RefSeq NM_005964, Swiss-Prot: P35580] silencing were synthesized by Sigma (St. Louis, MO, US). As negative control siRNA against firefly luciferase was used. Transfection of siRNA was performed by electroporation using Amaxa Nucleofector™ according to manufacturer's protocol. Functional assays were performed 72 h after transfection.

### Wound healing assay

For the analysis of directional migration, we made use of the wound healing assay. For the myosin II-B silencing experiments, cells were collected and electroporated as described above. After electroporation, 300'000 cells were seeded onto 6-well plate. After 72 hours a wound was made in the monolayer and every 3 hours until 15 hours after wounding, a picture was made. After the experiment, the diameter of the initial wound was measured and relative wound closure based on initial wound diameter, was calculated. Three independent experiments were performed and a Student t-test (α = 0,05) was used for statistical significance. For wound healing assay using pharmacological modulators Y27632 and KT5926, inhibiting Rho kinase activity and an inhibitor of myosin light chain kinase (MLCK) activity, respectively, cells were seeded onto 6-well plate and incubated for 72 hours until desired confluency was reached. Then, medium containing 2 μM KT5926 or 10 μM Y27632 and controls were added to the wells. Plates were incubated for one hour and a wound was scratched. Further steps were similar as described above.

### Matrigel invasion assay

To test invasion through extracellular matrix, we made use of the Matrigel invasion assay. Briefly, transwell chambers with polycarbonate membrane filters (8.0 μm pore size, 6.5 mm diameter, Costar, NY, US) were coated with 40 μL of matrigel solution (Becton Dickinson). BLM melanoma cells were added on top of the matrigel solution 60 hours post transfection. Conditioned medium of cultured CT5.3 myofibroblasts was added as a chemoattractant in the lower compartment. After 24 hours incubation, the remaining (invasive) cells at the lower surface of the filter were fixed with cold methanol and stained with 4',6'-diamidino-2-phenylindole (DAPI) (Sigma-Aldrich, Belgium). Invasive cells were scored by counting 30 fields per filter with a fluorescence microscope, at × 200 of magnification. Statistical significance was determined by the Student's t-test; p < 0.05 was considered significant.

### Immunohistochemical staining

5 μ thick paraffin slides of eight nodular melanomas were cut and pretreated with protease-1 (Ventana, France) for four minutes. Indirect immunohistochemical staining was performed with polyclonal antibodies directed against myosin II-B and P-cadherin for 30 minutes according to the manufacturer's protocol (Ventana, France) and by use of the automated Benchmark^® ^system (Ventana, France).

## Competing interests

The authors declare that they have no competing interests.

## Authors' contributions

KJ made substantial contributions to conception, design, acquisition and interpretation of data and wrote the manuscript. MVG and RF performed experiments and analyzed data. LB provided samples and analyzed data. BVH and ODW designed and revised the manuscript critically. MB has been involved in drafting the manuscript and revising it critically for important intellectual content. All authors read and approved the final manuscript.

## Supplementary Material

Additional file 1 **Downregulated calcium channels and associated proteins in BLM P-cad as indicated in the microarray experiment**. Overview of mRNA sequences that were considered to be downregulated in the microarray experiment concerning calcium signaling. The right column shows the identification of the probe set that was used during the microarray experiment. The gene title reflects the gene name that corresponds to the sequence that was differentially detected in both cell lines. The ratio(^2^logX) shows the numeric detection difference of a mRNA sequence between BLM LIE and BLM P-cad. Minus indicates a downregulation in BLM P-cad and the factor is of logarithmic scale. Click here for file
