# Supplementary figures and images for: P-cadherin counteracts myosin II-B function: implications in melanoma progression
Source: Mol Cancer. 2010 Sep 22;9:255. doi: 10.1186/1476-4598-9-255 (PMC2949802; doi:10.1186/1476-4598-9-255)

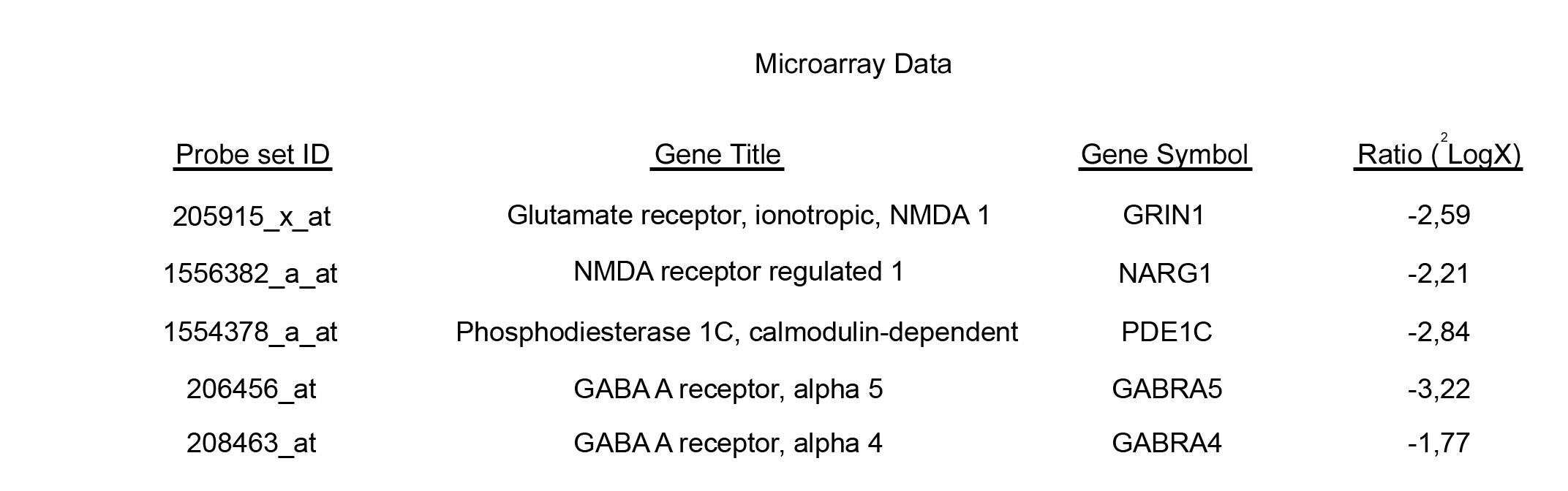

Supplement: Additional file 1 — Downregulated calcium channels and associated proteins in BLM P-cad as indicated in the microarray experiment. Overview of mRNA sequences that were considered to be downregulated in the microarray experiment concerning calcium signaling. The right column shows the identification of the probe set that was used during the microarray experiment. The gene title reflects the gene name that corresponds to the sequence that was differentially detected in both cell lines. The ratio(2logX) shows the numeric detection difference of a mRNA sequence between BLM LIE and BLM P-cad. Minus indicates a downregulation in BLM P-cad and the factor is of logarithmic scale. [file 1476-4598-9-255-S1.PNG]
